# Oral Health and Swallowing Problems

**DOI:** 10.1007/s40141-013-0026-x

**Published:** 2013-09-15

**Authors:** Michiko Furuta, Yoshihisa Yamashita

**Affiliations:** Section of Preventive and Public Health Dentistry, Division of Oral Health, Growth and Development, Faculty of Dental Science, Kyushu University, 3-1-1 Maidashi, Higashi-ku, Fukuoka, 812-8582 Japan

**Keywords:** Tooth loss, Denture, Saliva, Swallowing function, Swallowing problems, Dysphagia

## Abstract

Oral health impacts systemic health. Therefore, oral care is an important consideration in maintaining quality of life (QOL). Previously, maintenance and improvement of oral hygiene was considered essential for achieving oral health. In addition to oral hygiene, oral care in terms of oral function is now considered to maintain QOL. Ingestion of exogenous nutrients via the oral cavity is fundamental to the function of all higher animals, not only human beings. Chewing and swallowing processes are critical for normal food intake, and adequate saliva supply and oral care to allow proper functioning of these processes are indispensable for maintaining QOL. In this review, we will summarize the relationship between chewing and swallowing and effects of saliva secretion on these functions, and discuss methods to maintain ingestion of exogenous nutrients and prevent swallowing problems, especially in elderly people.

## Introduction

Oral health is defined as “a state of being free of mouth and facial pain, oral and throat cancer, oral infection and sores, birth defects such as cleft lip and palate, periodontal (gum) disease, tooth decay and tooth loss, and other diseases and disorders that limit an individual’s capacity in biting, chewing, smiling, speaking, and psychosocial wellbeing [1].” In short, the most important thing is to maintain oral function in terms of biting, chewing, smiling, speaking, and psychosocial wellbeing. The most common oral health problems include oral diseases such as dental caries and periodontal disease. These diseases are highly prevalent around the world, and nearly 100 and 90 % of adults have dental caries and periodontal disease, respectively [1, 2]. To achieve oral health, reduction and prevention of these diseases is required; therefore, oral care aiming at improvement and maintenance of oral hygiene to prevent these two major dental diseases should be promoted. Recently, proper oral hygiene is also thought to be essential for maintaining and improving systemic health, especially prevention of (1) aspiration pneumonia in the frail and elderly, and (2) post-operative infections [3–5]. Various combinations of measures and purposes of oral health maintenance (oral care) are summarized in Table 1.

**Table 1:** Four types of combinations of measures and purposes of oral health maintenance and related diseases or health conditions

Type	Measures	Purpose	Related problems
I	Maintaining and improving oral hygiene	Maintaining and improving oral health	Dental caries Periodontal diseases
II		Maintaining and improving systemic health	Aspiration pneumonia Prevention of post-operative infection
III	Maintaining and improving oral function	Maintaining and improving oral health	Malocclusion Hyposalivation
IV		Maintaining and improving systemic health	Swallowing problem Malnutrition Aspiration pneumonia

Both dental caries and periodontal disease are major causes of tooth loss, resulting in the fact that, in most countries, about 30 % of people aged 65–74 have no natural teeth [6]. Tooth loss reduces chewing ability. A longitudinal study showed that people who had incident of tooth loss were more likely to exhibit chewing difficulty than those without tooth loss [7]. In type III oral health maintenance, chewing is important to maintain the health condition of teeth and periodontal tissues through self-cleaning with adequate secreted saliva and mechanical stimulus on periodontal tissues. It is also thought to be closely related to swallowing, which is a part of oral function [8–11] as shown in Table 1. Furthermore, maintaining normal swallowing function in type IV oral health maintenance is important for systemic health, e.g., normal nutrient ingestion and preventing aspiration pneumonia, especially in the frail elderly.

In the present review, we will discuss how to maintain optimal swallowing function through oral health promotion to prevent (1) swallowing problems, i.e., dysphagia, and (2) systemic health effects based on the concepts of type IV oral health maintenance shown in Table 1.

## Chewing and Swallowing

Chewing, oral food transport, and swallowing is a continuum [10]; these processes, taken together, are often considered to represent the totality of the feeding process [12]. We coordinate the acts of chewing and intraoral food transport with swallowing [10, 11]. The act of chewing is considered to influence the timing of food transport and swallow initiation [11].

Chewing serves several functions including breakdown of large food particles into small pieces and lubricating and softening of food particles into a bolus conducive to swallowing [13]. If the food particles already meet the internal criteria for swallowing, the particles will be simply transported towards the pharynx for swallowing [12]. Chewing occurs by the action of the teeth, masticatory muscles, temporomandibular joint, and the tongue. When food is ingested into the oral cavity, the incisors break it off and the posterior teeth grind it. The jaw and tongue move by the help of masticatory muscles for chewing. The tongue surface rises upwards and travels backward during jaw closing, and reaches its maximum superior position in the intercuspal phase [14, 15]. As the jaw opens, the tongue surface continues to travel forwards and also downwards, to reverse its direction in relation to the maximum opening [14, 15]. The tongue movement plays a role in collecting and transporting the food bolus from the oral cavity into the pharynx [10, 12, 14, 16].

The quality of chewing can be evaluated in terms of “masticatory performance”, which is defined as the percentage of particle size distribution of food when chewed for a given number of strokes [17]. Masticatory performance reflects the capacity to reduce the size of food particles and the number of chews necessary to render food ready for swallowing [18, 19]. It is affected by the number of teeth in functional occlusion [20–22], the maximal biting force [23, 24], and denture wearing [25]. The salivary flow rate also affects masticatory performance, which declines with a reduction in salivary secretion [13].

## Effect of Tooth Loss and Denture Wearing on Swallowing Problems

### Effect of Tooth Loss

Chewing ability is dependent on the number of remaining teeth [20–22]. Thus, tooth loss is expected to indirectly disturb the coordinated execution of pre-swallow and swallowing behaviors. It has been shown that elderly people with reduced numbers of functional units, defined as any opposing natural or prosthetic tooth pair, complain of difficulty in chewing and swallowing [26]. Okamoto et al. [27] investigated the relationship between the number of remaining teeth, bite force, and swallowing problems assessed by the 30-mL water swallow test in 3,663 elderly people who were living independently. A positive correlation was observed between the number of remaining teeth and maximum bite force. They also reported that the adjusted odds ratios for 0–13 or 14–24 remaining teeth to 25–32 remaining teeth for swallowing problems were 2.04 [95 % confidence interval (CI) 1.60–2.60)] and 1.31 (95 % CI 1.02–1.70), respectively.

It is believed that tooth loss influences the oral preparatory stage of swallowing. There are three main stages in deglutition: oral, pharyngeal, and esophageal stages. Furthermore, the oral stage is split into two substages: an oral preparatory stage in which a bolus is formed through mastication and positioned for transport towards the pharynx or the liquid bolus cupped by the tongue in readiness for transit, and an oral transit stage in which the bolus is moved back through the oral cavity for passage to the pharynx with a front-to-back squeezing action, performed primarily by the tongue [28–30]. Tooth loss decreases chewing ability and causes difficulties in forming the bolus. Bolus size was reported to increase with an increasing number of missing teeth, with larger boluses potentially interfering with optimal swallowing [31]. Moreover, in people with compromised dentition, a poorly degraded bolus was swallowed in spite of the number of chewing cycles being increased [32, 33]. A poorly degraded bolus may be difficult to transport smoothly and efficiently into the pharynx, thus leading to additional swallowing abnormalities in the oral preparatory stage.

### Effects of Denture Wearing

A denture is a prosthesis that replaces one or more natural teeth. Wearing dentures in order to maintain appropriate mandible position and proper occlusion has been proposed as important for smooth swallowing in elderly individuals [34]. Fukai et al. [35] investigated the relationship between the number of functional teeth (defined as the teeth with functionally adequate mastication), denture use, and subjective swallowing problems in 5,643 residents aged 40–89 years. Subjective swallowing problems were self-assessed by reporting any kind of subjective impairment to eating function such as biting difficulty, swallowing difficulty caused by tooth loss, no fitted dentures, or other oral impairments. They concluded that residents with fewer functional teeth or no denture use were likely to have subjective swallowing problems, and suggested that, even if people have few teeth, denture wearing contributes to prevention of swallowing problems.

We examined the relationship between the number of teeth, denture wearing, and swallowing function in 286 elderly people aged 60 years and older (mean age 84.5 ± 7.9 years) living at home and receiving home care services [36••]. In this study, swallowing function was examined by cervical auscultation, a non-invasive method of listening with a stethoscope to the sounds of swallowing 3 mL of water during the pharyngeal phase, based on the method of Zenner et al. [37] with minor modifications. When breath sounds after swallowing material were clear, we considered swallowing function normal. When stridor, coughing, or throat clearing was heard after swallowing material or when swallowing was repeated, we evaluated this as impaired swallowing function (i.e. swallowing disorder or dysphagia). Swallowing impairments were found in 31.1 % of all participants. When the oral health status was categorized as 20 or more teeth with dentures, swallowing problem was found in 20.0 % of those who had ≥20 teeth with dentures, 26.5 % of those who had ≥20 teeth without dentures, 30.0 % of those who had 0–19 teeth with dentures, 38.9 % of those who had 0–19 teeth without dentures, 27.5 % of those who had 0–9 teeth with dentures, and 57.7 % of those who had 0–9 teeth without dentures. Additionally, when the effect of denture wearing on swallowing function in edentulous persons was examined, 10 of 15 edentates without dentures (66.7 %) had difficulty swallowing, as did 29 of 101 edentates with dentures (28.7 %). A previous study reported that laryngeal penetration, usually due to neuromuscular disorder, occurs with much greater frequency in edentulous elderly people who are not wearing dentures than in dentate subjects, but laryngeal penetration was reduced in edentulous elderly people when wearing dentures [38]. Although the potential effect of laryngeal penetration on health is unclear, it has been described that laryngeal penetration occurs when the coordinated movements of swallowing cannot continue due to some interference [29].

While some studies [34–36••, 38–40] have suggested that denture wearing had positive effects on swallowing function, conversely, one study [41] indicated that denture wearing may have negative effects on swallowing function, especially the oral transit time (OTT) and oropharyngeal swallow efficiency (OPSE). OTT is the time from the moment at which the major bolus in the oral cavity begins to move rearwards to the moment at which the front end of the bolus arrives at the point of crossing between the lower edge of mandibular ramus and the tongue base. OPSE is the value obtained by dividing the fraction of bolus swallowed through the esophagus by the total time taken to pass the oropharynx, and aspiration is the passing of food through the vocal fold [42]. Son et al. [41] showed that OTTs were shortened and OPSE increased when dentures were removed compared to when dentures were worn. They discussed that the delay in OTTs and the decreases in OPSE observed after dentures were worn might be considered attributable to changes in masticatory performance or sensation within the oral cavity due to denture wearing. However, it seems that this finding might be influenced by the quality of denture, which they did not assess. Wearing ill-fitting dentures, particularly in the case of a complete denture, may interfere with achieving sufficient occlusal contact. Insufficient occlusal contact reduces masticatory performance and causes impairment of swallowing activity.

Two studies have shown that the quality of the denture influenced swallowing function. Monaco et al. [43•] investigated whether an ill-fitting denture increases swallowing duration time in elderly people using surface electromyography, which assesses certain aspects of complex muscle activity in deglutition. Swallowing duration ranges from 0.80 to 1.60 s [44, 45], and this normal duration is achieved by 12 years of age. Swallowing duration range does not change significantly until 70 years of age and increases after that [46–49]. They found that the mean swallowing time in participants with old, poorly fitting dentures was 1.84 s, while new, properly fitting dentures needed a swallowing time of 1.28 s [43•]; swallowing duration with ill-fitting dentures was significantly longer than with properly fitting dentures. They suggested that the quality of the denture is more important in the impairment of swallowing duration than individual neuromuscular efficiency and ageing [43•]. In addition, Yoshida et al. [39] examined rehabilitation hospital inpatients whose old dentures were adjusted by repairing or relining on the day of the study and showed that pharyngeal transit time was shorter when wearing dentures than when not wearing dentures. Because prolongation of pharyngeal transit time increases the risk of aspiration [50, 51], repair of ill-fitting dentures may decrease the risk of aspiration. Future studies investigating the effect of wearing denture on swallowing function should consider the quality of the denture and examine further interventions, as most studies employed cross-sectional designs.

Wearing dentures in those who have lost teeth may have a beneficial effect on not only swallowing function but also cognitive function. One cohort study showed that men who had inadequate natural masticatory without wearing dentures had a 91 % greater risk of dementia than those with adequate natural mastication [52]. Retaining adequate masticatory function through maintaining and improving oral hygiene and oral function (e.g., oral care and use of dentures) may reduce both swallowing problem and risk of dementia.

## Effect of Saliva Secretion on Swallowing Problem

Functions of saliva include cleansing of oral cavity, solubilization of food substances, bolus formation, facilitation of mastication and swallowing, food and bacterial clearance, dilution of detritus, lubrication of mucosa, and facilitation of speech [13]. Decrease in saliva secretion (i.e. dry mouth, or xerostomia) is frequently accompanied by swallowing problems and is common in elderly people [53], with incidence ranging from 20 % to as high as 60 % [54–58]. The dryness of the mouth affects both the oral preparatory and oral phases of swallowing, and can lead to impaired bolus formation and oropharyngeal bolus transport [59]. Additionally, severe dental diseases such as dental caries and periodontal diseases which result in decreased saliva secretion can lead to pain and impair food preparation [60].

Considering the role of saliva for swallowing, swallowing disorders in people with dry mouth can be managed with oral moisturizers, lubricants, and careful use of fluids during eating [56]. Intrinsically, increasing salivary flow may help them swallow smoothly. Kakudate et al. [61] reported that elderly people with tooth brushing frequency less than twice per day were more likely to have dry mouth. Additionally, Papas et al. [62] showed that tooth brushing increased salivary flow in people with medication-induced dry mouth. It is suggested that tooth brushing may activate impulses to both major and minor residual salivary tissues following stimulation of oral and pharyngeal regions, causing salivation [62, 63]. In other words, mechanical stimulation of the salivary glands during tooth brushing can promote the discharge of saliva [61]. It is considered that tooth brushing helps to maintain and improve not only oral hygiene but also oral function through an increased salivary flow rate. However, we need to consider the possibility of developing a more effective method for mechanical stimulation of salivary glands than tooth brushing.

On the other hand, an excessive production of saliva (hypersalivation) may produce swallowing problems caused by aspiration of saliva [64]. Hypersalivation is induced by inflammation in the oral-pharyngeal regions, neurological disorders such as Parkinson’s disease and epilepsy, or medicine such as pilocarpine and cholinesterase [65]. In particular, neurological disorders results in swallowing problem and leads to excessive pooling of saliva in the oral cavity and the unintentional loss of saliva from the mouth, i.e. drooling of saliva [66]. Drooling can impair masticatory function [66], and thus it may cause a vicious cycle of swallowing problems. Since reduced or increased salivary secretions leads to swallowing problems, suitable amounts of saliva would retain normal swallowing function.

## Oral Care for Swallowing Disorders

Yoneyama et al. [4] demonstrated that oral care comprising maintenance and improvement of oral hygiene reduced the risk of pneumonia in older patients receiving nursing care. However, maintenance and improvement of oral hygiene may not be enough to achieve good oral health; the contribution of oral function to oral health should also be considered. Normal swallowing is closely related to chewing ability with normal dentition and ample saliva secretion [11, 13, 59, 60]. Healthy chewing and swallowing may prevent a decline in performing the basic activities of daily living. Recently, we revealed that tooth loss was directly related to deterioration of swallowing function and subsequently to the ability of nutritional intake and then to the basic activities of daily living [36••]. These findings indicate that oral care, provision of dentures, prevention of tooth loss by treatment for dental caries or periodontal disease, and swallowing training may have positive effects on general health in frail older people.

On the other hand, people with swallowing problems, especially elderly people having physical disabilities, appear to be at increased risk of poor oral health, for example, because of an inability to chew because of few remaining teeth or wearing ill-fitting dentures. While oral care is needed to maintain and improve oral health, people with swallowing problems may have difficulty in performing oral care. When we investigated the relationship between oral care and swallowing problems in 231 participants aged 60 years or older who received home nursing care [36••], people with swallowing problems were more likely to fail in maintaining seated posture, cervical movement, keeping the jaw open, holding water in the oral cavity, and gargling compared to those with normal swallowing function (Table 2). As people with swallowing problems had more severe cognitive impairment and more malnutrition than those with normal swallowing ability, they participated in fewer activities of daily living; in other words, they had physical disabilities. Therefore, it was likely that they had trouble receiving oral care. However, if adequate oral care is not provided, not only oral health but also the general health including swallowing will steadily worsen. In cases of difficulty in providing oral care, oral care with management of the swallowing problems by the coordinated expertise of a number of health care professionals might be effective.

**Table 2:** Swallowing problems and oral care-related factors

	Normal (*n* = 165)	Swallowing problems (*n* = 76)	*p* value
Oral care			0.951
Do	156 (94.5)	72 (94.7)	
Do not	9 (5.5)	4 (5.3)	
Independence in oral care^a^			<0.001
Yes	125 (80.1)	41 (56.9)	
No (dependent)	31 (19.9)	31 (43.1)	
Refusal of oral care^a^			0.014
No	150 (96.2)	63 (87.5)	
Yes	6 (3.8)	9 (12.5)	
Oral care-related factors			
Tube feeding			0.010
No	165 (100.0)	73 (96.1)	
Yes	0 (0.0)	3 (3.9)	
Seated posture			<0.001
Possible	165 (100.0)	66 (86.8)	
Impossible	0 (0.0)	10 (13.2)	
Cervical movement			<0.001
Possible	163 (98.8)	62 (81.6)	
Impossible	2 (1.2)	14 (18.4)	
Keeping jaw open			0.007
Possible	161 (97.6)	68 (89.5)	
Impossible	4 (2.4)	8 (10.5)	
Holding water in oral cavity			<0.001
Possible	160 (97.0)	58 (76.3)	
Impossible	5 (3.0)	18 (23.7)	
Gargling			<0.001
Possible	152 (92.1)	50 (65.8)	
Impossible	13 (7.9)	26 (34.2)	

*n *(%), Chi square test or Fisher’s exact test

^a^Participants who perform oral care, *n* = 228

## Conclusion

Swallowing problems closely related to poor oral health have a negative impact on the quality of life of those suffering from them. Preventing tooth loss, wearing properly fitting dentures when teeth cannot be saved, and promoting normal saliva secretion may provide beneficial effects on swallowing function and subsequently on the maintenance and improvement of systemic health. It is suggested that oral care plays an important role in maintaining and improving not only oral health but also systemic health.
